# Molecular Epidemiology of Human Rhinovirus From 1-Year Surveillance Within a School Setting in Rural Coastal Kenya

**DOI:** 10.1093/ofid/ofaa385

**Published:** 2020-08-27

**Authors:** Martha M Luka, Everlyn Kamau, Irene Adema, Patrick K Munywoki, Grieven P Otieno, Elijah Gicheru, Alex Gichuki, Nelson Kibinge, Charles N Agoti, D James Nokes

**Affiliations:** 1 Epidemiology and Demography Department, KEMRI-Wellcome Trust Research Programme, Centre for Geographic Medicine Research – Coast, Kilifi, Kenya; 2 Department of Public Health, Pwani University, Kilifi, Kenya; 3 School of Life Sciences and Zeeman Institute for Systems Biology and Infectious Disease Epidemiology Research (SBIDER), University of Warwick, Coventry, UK

**Keywords:** human rhinovirus, Kenya, molecular epidemiology, school-going children, transmission

## Abstract

**Background:**

Human rhinovirus (HRV) is the most common cause of the common cold but may also lead to more severe respiratory illness in vulnerable populations. The epidemiology and genetic diversity of HRV within a school setting have not been previously described. The objective of this study was to characterize HRV molecular epidemiology in a primary school in a rural location of Kenya.

**Methods:**

Between May 2017 and April 2018, over 3 school terms, we collected 1859 nasopharyngeal swabs (NPS) from pupils and teachers with symptoms of acute respiratory infection in a public primary school in Kilifi County, coastal Kenya. The samples were tested for HRV using real-time reverse transcription polymerase chain reaction. HRV-positive samples were sequenced in the VP4/VP2 coding region for species and genotype classification.

**Results:**

A total of 307 NPS (16.4%) from 164 individuals were HRV positive, and 253 (82.4%) were successfully sequenced. The proportion of HRV in the lower primary classes was higher (19.8%) than upper primary classes (12.2%; *P* < .001). HRV-A was the most common species (134/253; 53.0%), followed by HRV-C (73/253; 28.9%) and HRV-B (46/253; 18.2%). Phylogenetic analysis identified 47 HRV genotypes. The most common genotypes were A2 and B70. Numerous (up to 22 in 1 school term) genotypes circulated simultaneously, there was no individual re-infection with the same genotype, and no genotype was detected in all 3 school terms.

**Conclusions:**

HRV was frequently detected among school-going children with mild acute respiratory illness symptoms, particularly in the younger age groups (<5-year-olds). Multiple HRV introductions were observed that were characterized by considerable genotype diversity.

Human rhinovirus (HRV) is a common viral respiratory pathogen [1] associated with the common cold [2, 3], lower respiratory tract infections [4], and asthma [5]. Although the majority of HRV cases are mild and self-limiting [6], they contribute to substantial economic losses through missed school and workdays [6, 7]. HRV is a common reason for prescribing antibiotics [8], potentially contributing to antibiotic resistance. The virus is transmitted via inhalation of contaminated aerosols, for example, during close contact with infected persons, or self-inoculation via touching contaminated surfaces or objects [6, 9]. HRV has a median incubation period (range) of 2 (1–7) days and a symptom duration of 7–14 days [10]. Viral shedding can occur for up to 2 (range, 1–3) weeks [11]. Children, the elderly, and persons with preexisting respiratory conditions bear the highest HRV burden [12–14].

HRV is a positive-sense, single-stranded RNA virus, classified under the genus *Enterovirus* (family *Picornaviridae*), with a genome ~7.2 kb long. It is characterized by high genetic and antigenic diversity [15], frustrating vaccine development efforts. There are 169 HRV genotypes distributed across 3 species: HRV-A (80), HRV-B (32), and HRV-C (57) genotypes [16]. Molecular classification of HRV into genotypes is based on the nucleotide sequence of either the 5’ noncoding region, VP1, or the VP4/2 genome region [17–19].

Children constitute a significant susceptible population that support the transmission and persistence of HRV in populations [20]. In Africa, little has been done to investigate the patterns and mechanisms of transmission of HRV in the school setting and to understand the extent to which school settings contribute to HRV transmission in the community. This is despite that school children have high contact rates compared with other age groups in the community [21–23]. Design of effective intervention strategies against HRV will be supported by improved knowledge of transmission dynamics of HRV in different social networks and population structures [6, 24]. This study investigated HRV infections in a school setting in rural coastal Kenya by sequence analysis of the VP4/2 junction to describe the frequency, diversity, and temporal occurrence of HRV.

## METHODS

### Study Area and Design

The study was conducted in a rural school located within the Kilifi Health and Demographic Surveillance System (KHDSS) in Kenya [25] to characterize the occurrence of respiratory viruses. The study design is described in detail elsewhere [26]. Briefly, the school offers both early childhood development education and primary school education. Pupils and teachers from all classes were enrolled in the study, which took place between May 2017 and April 2018. Pupils were divided into 2 main groups: the lower primary, comprised of day care, kindergarten (KG) levels 1–3, and grade 1 (n = 5 classes; age range, 3–12 years); and the upper primary, comprised of grades 2–8 (n = 7 classes; age range, 7–20 years).

Nasopharyngeal swabs were collected when a pupil or a teacher had at least 1 of the following acute respiratory illness (ARI) symptoms: cough, sore throat, or runny nose. Students documented the ARI symptoms they experienced in a daily diary. Class teachers recorded symptoms for the lower primary group. A maximum of 8 samples per class was collected from the lower primary group per week, while a maximum of 4 samples per class was collected from the upper primary group per week. A maximum of 3 samples was collected from the teachers per week. We collected more samples from the lower primary due to the perceived critical role of this age group in childhood infectious diseases and hence the need to reduce the level of uncertainty in the estimated risk in this age group. Samples were collected in viral transport media (VTM) and transported in cool boxes to the KEMRI-Wellcome Trust Research Programme laboratory where they were stored at –80°C before screening. Sampling was suspended during school holidays: August, November, and December 2017 and after April 6, 2018, which marked the end of the study.

### Patient Consent Statement

This article reports on samples collected from 2 studies: a school [26] and a community ARI surveillance study [27]. An informed written parental consent for persons under the age of 18 years or individual consent for adults was obtained before sample collection. For the school cohort, consent was obtained at the beginning of the study, with new students and those not initially enrolled allowed to join in the second and third terms. In addition, children whose parents consented were asked for individual assent to participate. Ethical approval was provided by the KEMRI-Scientific Ethics Review Unit (KEMRI-SERU #3332 and #3103) and the University of Warwick Biomedical and Scientific Research Ethics Committee (BSREC #REGO_2016-1858 and #REGO-2015–6102).

### RNA Extraction and Real-time Reverse Transcription Polymerase Chain Reaction

RNA was extracted from 140 µL of the collected samples using the QIAamp 96 Virus QIAcube HT kit (Qiagen, Manchester, UK), according to the manufacturer’s instructions. Samples were screened for 15 virus targets [26]—HRV, respiratory syncytial virus (A and B), human coronaviruses (OC43, NL63, and E229), influenza (A, B, and C), parainfluenza (1–4), adenovirus and human metapneumovirus—using in-house multiplexed real-time reverse transcription polymerase chain reaction (rRT-PCR) with a QuantiFast Multiplex RT-PCR kit (Qiagen, Manchester, UK) [28–30]. A sample was considered HRV positive if the rRT-PCR cycle threshold (*Ct*) value was <35.

### VP4/2 Amplification and Sequencing

VP4/2 sequencing was used to assign species and genotypes. A genomic region of ~549 nucleotides and consisting of a hypervariable region of the 5’UTR, the complete VP4, and the partial VP2 gene region was amplified for all HRV-positive samples using a One-Step RT-PCR kit (QIAGEN) as previously described [31, 32]. PCR products were purified using the MinElute PCR purification kit (Qiagen, Manchester, UK) and sequenced with the respective forward and reverse primers in a BigDye terminator, version 3.1 (Applied Biosystems, Foster City, California, USA), and analyzed in an ABI 3130xl genetic analyzer.

### Sequence Analysis

Raw sequence reads were quality-checked, trimmed, edited, and assembled to contigs of 420 nucleotides using Sequencher, version 5.4.6 (www.genecodes.com). Alignments were prepared using MAFFT, version 7.271 [33]. Full exploratory recombination scans were done using RDP4 software [34]. IQ-TREE, version 1.6.0 [35], was used to estimate the best fitting model and infer maximum likelihood (ML) trees. Phylogenetic trees were generated with bootstrapping of 1000 iterations. Pairwise nucleotide p-distances were calculated using MEGA, version 7.0.21 [36]. Genotype assignment was based on phylogenetic clustering (bootstrap value >80%) on ML trees and pairwise genetic distances to prototype strains (https://www.picornaviridae.com/enterovirus/prototypes/prototypes.htm) as proposed (10.5% for HRV-A, 9.5% for HRV-B, and 10.5% for HRV-C) [17, 18].

Intratype diversity for genotypes with ≥10 sequences was studied by visualization of the number of nucleotide substitutions to the index sequence for each type. The substitution rate of the VP4/2 coding region in HRV had previously been estimated as 7 × 10^–4^ – 4 × 10^–3^ substitutions/site/year [37]. Using the upper evolutionary rate value translated to 1.68 nucleotide substitutions per year across the sequenced 420 nt segment. We therefore defined an intratype variant as a sequence with >2 nucleotide differences from the index sequence. An intratype variant had to be observed in at least 2 sequences to increase confidence that this was not the result of sequencing error.

### Data Analysis

Data analysis was conducted using STATA, version 13 (STATA Corp, TX, USA), and R, version 3.6.1 (CRAN R Project). Categorical variables were summarized using frequencies and percentages. The HRV proportion for each class and respective 95% CIs were defined as the number of HRV-positive samples out of the total number of samples tested per class. The chi-square test for trend was used to check for linear trend in HRV proportion with an increasing hierarchy of classes in the school (from day care to teaching staff). Spearman’s rank correlation coefficient was run to determine correlation between age and Ct value.

### Definition of Terms

We defined “persistence” as the continued occurrence of the same genotype within the same school term. Detection of a genotype in a subsequent school term was considered a genotype recurrence. We defined “frequent” genotypes as those that occurred in ≥5 samples, from >2 individuals, and further investigated their temporal occurrence and persistence. We defined “individual HRV re-infection” as the acquisition of a new genotype or the detection of a previously acquired genotype in a subsequent school term. Individual detection with the same genotype in consecutive samples was considered a continuing infection.

### Sequence Data Availability

Sequences generated by this study are available in GenBank under accession numbers MT177659–MT177911.

## RESULTS

### Baseline Characteristics and HRV Detection

A cohort of 371 individuals (358 pupils and 13 teachers) was followed up for the development of ARI symptoms between May 2017 and April 2018. The total number of samples collected was 1859, of which 307 (16.5%) tested positive for HRV. Twenty-six of the HRV-positive samples had co-infections with other viruses. Of the 1552 HRV-negative samples, only 115 tested positive for at least 1 virus target (Supplementary Table 1). The HRV positives were collectively from 164 individuals: 160 pupils and 4 teachers. The mean age of the HRV-positive pupils was 9.4 years, with a standard deviation of ±3.9. The most common ARI symptom recorded among the HRV-positive cases (single sampling events) was nasal discharge (n = 278; 90.6%), followed by cough (n = 226; 73.6%) and sore throat (n = 62; 20.2%). There was no notable difference between symptoms in HRV-positive and negative cases (*P* > .05). Only a small proportion of those detected with HRV could identify a household member with ARI-like symptoms (n = 43, 14.0%). Among the household members identified, 26/43 (60%) were school-going siblings, with 18 (69%) of these attending the same school and the other 8 (31%) a different school (Table 1).

**Table 1.** Baseline Characteristics of the HRV-Positive Cases at a Rural Kenyan School Sampled Throughout 3 Terms From May 2017 to April 2018

Characteristic	Categories	No. Pos	% Pos	No. Neg	% Neg	Total
Age (pupils)	≤5 y	78	22.4	270	77.6	348
	6–10 y	137	17.6	641	82.4	778
	11–17 y	82	12.4	578	87.6	660
	≥18 y	2	5.9	32	94.1	34
	Unspecified	3	42.9	4	57.1	7
Age (teachers)	≥18 y	5	15.6	27	84.4	32
	Total	307	16.5	1552	83.5	1859
Gender	Male	169	18.8	731	81.2	900
	Female	138	14.4	821	85.6	959
Symptoms in the last 2 wk						
Cough	Yes	226	16.6	1137	83.4	1363
	No	81	16.4	413	83.6	494
Nasal discharge	Yes	278	17.0	1357	83.0	1635
	No	29	13.1	193	86.9	222
Sore throat	Yes	62	16.2	321	83.8	383
	No	245	16.6	1227	83.4	1472
Household members						
Other persons in HH with symptoms of ARI?	Yes	43	14.2	260	85.8	303
	No	215	17.4	1024	82.7	1239
	Don’t know	49	15.5	268	84.5	317
Are they in school? (n = 43)	Yes	26	16.1	136	84.0	162
	No	17	12.1	124	87.9	141
	Unspecified	264	17.0	1292	83.0	1556

Abbreviations: % Neg: percent negative; % Pos: percent positive; ARI, acute respiratory infection; HH, household; HRV, human rhinovirus; No. Neg: number negative; No. Pos: number positive.

**Figure 1.**
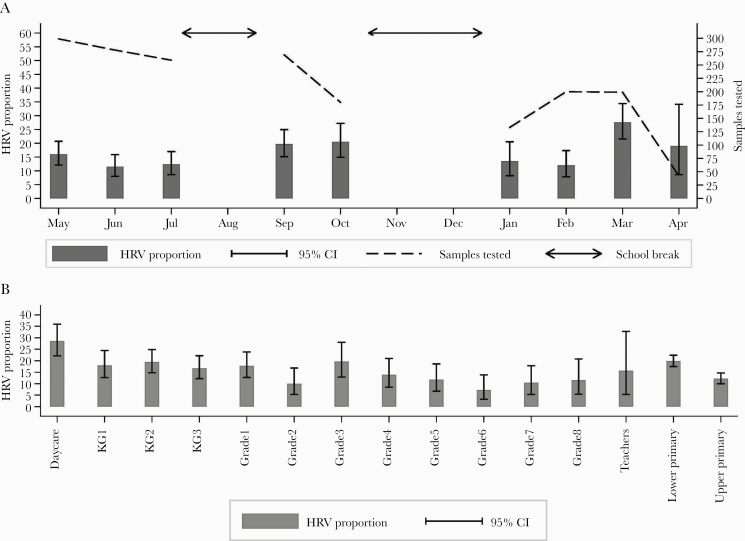
Patterns of HRV infections within the school over 1 year. A, Month-by-month HRV proportion with respective 95% confidence intervals and number of samples tested. B, Class-specific HRV proportion with respective 95% confidence intervals. The 2 bars furthest to the right are aggregated proportions of lower primary and upper primary groups. Abbreviation: HRV, human rhinovirus.

HRV circulation was detected throughout the 3 school terms under observation. Seasonal variations of HRV infections could not be identified due to breaks in sample collection during the school holidays. The lower primary had a higher HRV proportion compared with upper primary (19.8% vs 12.2%; *P* < . 001) (Figure 1). Spearman’s *rho* indicated no correlation between Ct values and age (*rho *= .03; *P *= .615).

### HRV Diversity

**Figure 2.**
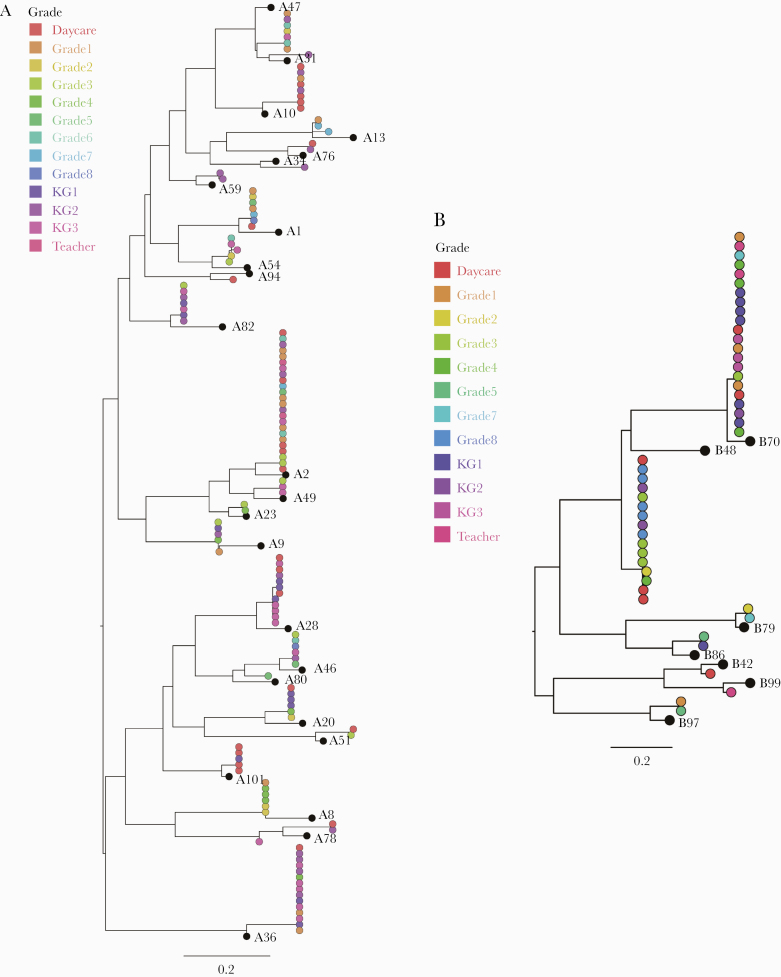

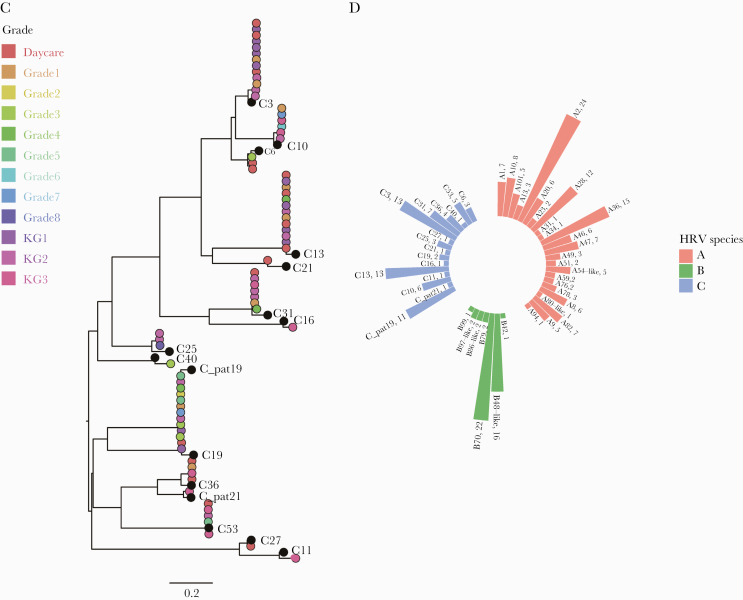
Phylogenetic analysis and genotype assignment of generated HRV sequences. Species-specific maximum likelihood trees of (A) HRV-A, (B) HRV-B, and (C) HRV-C. The tip shapes are colored by the school class of the individual. The scale bars represent nucleotide substitutions per site. The black tips represent the prototype sequence of respective strain. D, A circular bar plot showing the frequencies of HRV genotypes identified. The bars are colored by HRV species and the tips are labeled by HRV type and frequency. Abbreviation: HRV, human rhinovirus.

Amplification and sequencing of the VP4/2 genomic region were attempted on all HRV-positive samples, resulting in 82.4% (253/307) success. The unsuccessful samples either failed to amplify or had poor sequence quality. The resulting sequences were classified into 47 HRV genotypes: 24 HRV-A genotypes, 7 HRV-Bs, and 16 HRV-Cs. HRV-A was the most common species (134/253; 53.0%), followed by HRV-C (73/253; 28.9%) and HRV-B (46/253; 18.2%). Some sequences violated the previously proposed genotype assignment thresholds (Supplementary Table 2). These were included in the analysis and classified with a suffix “-like” to the most similar known genotype (eg, B48-like). The most frequent genotypes were A2 (24/253; 9.5%), B70 (22/253; 8.7%), A36 (16/253; 6.3%), and B48-like (16/253; 6.3%) (Figure 2). No recombination events were identified.

### Temporal Occurrence and Spatial Clustering Patterns

**Figure 3.**
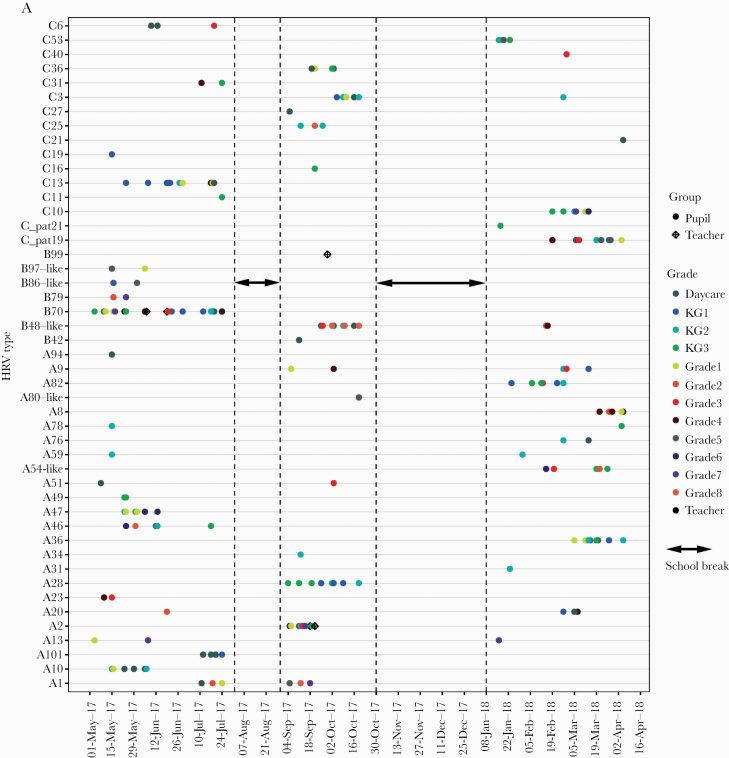

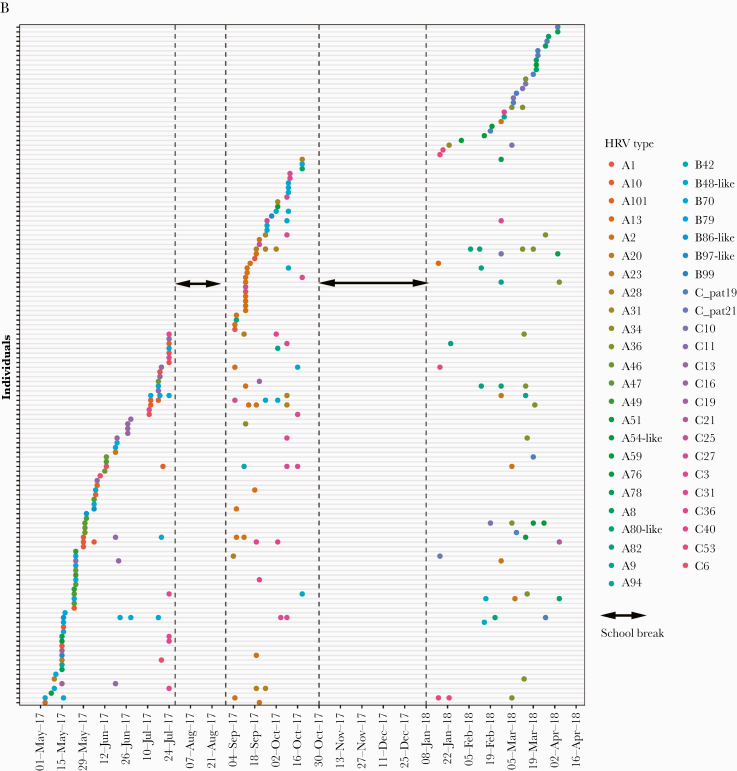

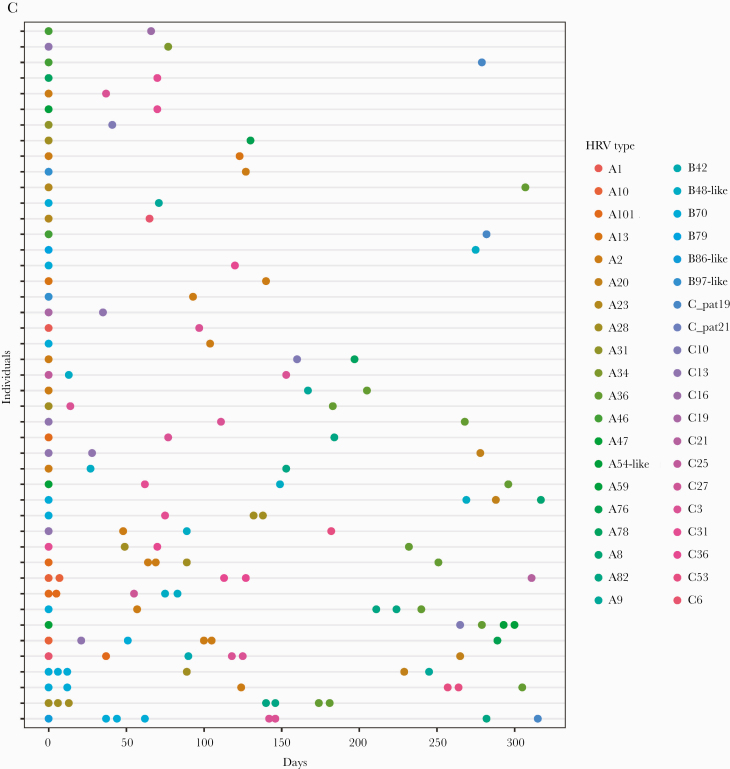
The temporal occurrence and infection patterns of HRV. A, The temporal occurrence of all HRV genotypes across the 1-year study period. B, Individual infection patterns across the 1-year period. The individuals are ordered by date of first HRV infection. C, Number of days to a subsequent HRV case for individuals who were re-infected during the study period. All individual first infections are dated day 0. The individuals are ordered by number of HRV-positive samples, with the individual with the most positive samples appearing at the bottom. Abbreviation: HRV, human rhinovirus.

Numerous genotypes circulated simultaneously in the school, with 22, 15, and 19 unique genotypes observed in term 1, term 2, and term 3, respectively. Nine genotypes recurred during the study period. Of the 22 that occurred in term 1, 2 re-occurred in term 2, and 4 in term 3, whereas of the 15 observed in term 2, 3 recurred in term 3. No genotype was observed across all 3 school terms. Four of the recurring genotypes (A13, A59, B48, C3) were detected in the same class (Figure 3A).

**Figure 4.**
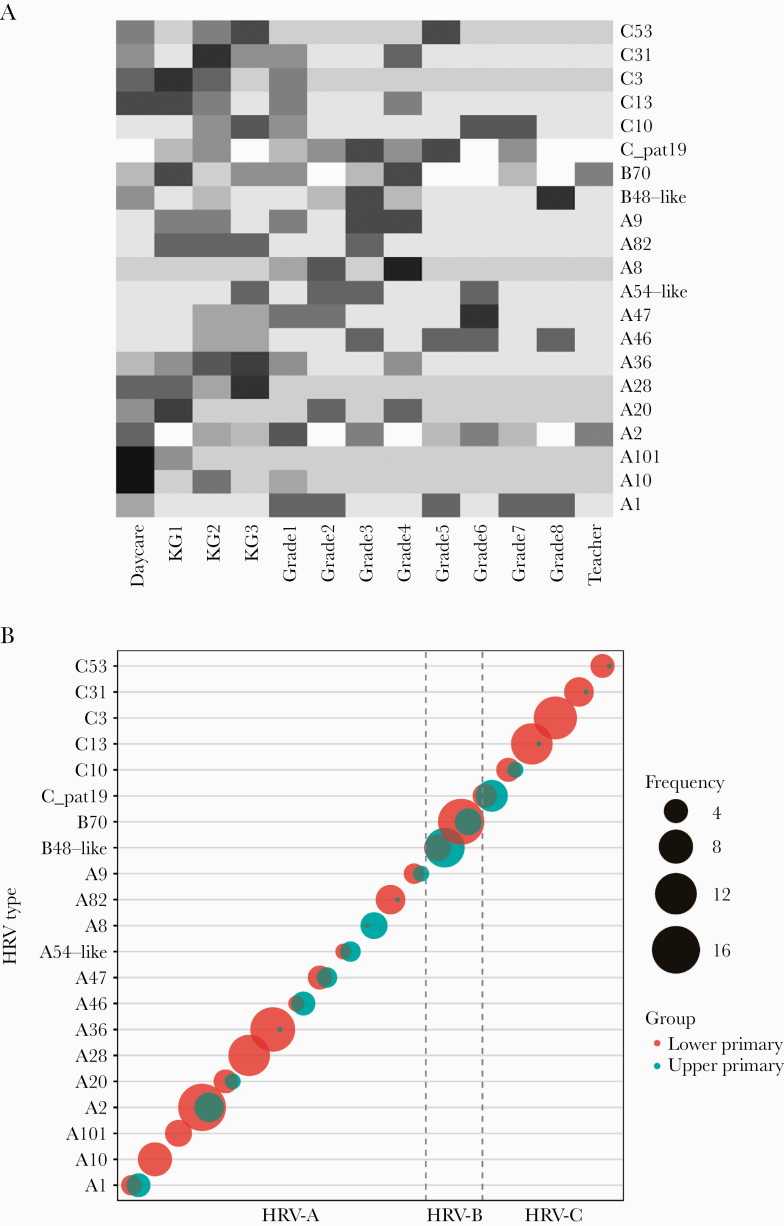
Distribution of genotypes across the school. A, Heatmap showing the distribution of frequent genotypes across the school classes. The intensity of the color correlates to the frequency of samples. Color intensity has been scaled to correct for sampling bias between the lower primary and upper primary classes. B, Distribution of frequent genotypes between the upper and lower primary groups. The sizes of the circles correlate to the number of samples and the color to the respective group: either lower or upper primary. Abbreviation: HRV, human rhinovirus.

Twelve genotypes were observed as singletons (ie, present in a single sample/individual). We observed that frequent genotypes (n = 21) circulated averagely for 28 days (median, 23 days). Five of the frequent genotypes recurred in a subsequent school term. The longest persisting genotype was B70 (n = 22 samples), seen in 81 days. Genotype A2 (n = 24 samples) was similarly frequent but persisted for only 16 days. Among the frequent genotypes, none was limited to the upper primary group, whereas A10, A28, A101, and C3 were observed only in the lower primary group. However, no frequent genotype was limited to only 1 school class (Figure 4).

### Individual Infection Patterns

Of the 164 HRV-positive individuals, 62 (37.8%) contributed >1 positive sample. Three pupils, all from the lower primary group, presented with the most HRV-positive samples (n = 8) per person. The 253 successfully sequenced samples were collectively contributed by 144 individuals. Repeat HRV detections (n = 109) were a combination of persistent infections (24/109) and re-infections (85/109). The number of genotypes per person ranged from 1 to 5. About two-thirds of the individuals (98/144; 68.1%) had only 1 HRV genotype across the study period. Overall, the highest HRV diversity per person (5 genotypes) was observed in 3 pupils, all from the lower primary classes (Table 2). Time to re-infection varied greatly (13–307 days), with a median of 77 days. However, no individual was re-infected with the same genotype across the study period (Figure 3).

**Table 2.** Human Rhinovirus Detection and Genotyping Summary Among Individuals

Distribution of HRV Among Individuals	Frequency of HRV-Positive Samples/Person	No. of Individuals	Percentage
	1	102	62.2
	2	31	18.9
	3	13	7.9
	4	5	3.0
	5	3	1.8
	6	4	2.4
	7	3	1.8
	8	3	1.8
	Total	164	100
Distribution of Sequenced Samples Among Individuals	Frequency of Sequenced Samples/Person	No. of Individuals	Percentage
	1	96	66.7
	2	23	16.0
	3	9	6.3
	4	5	3.5
	5	5	3.5
	6	4	2.8
	7	1	0.7
	8	1	0.7
	Total	144	100
Diversity of HRV	No. of Unique Types/Person	No. of Individuals	Percentage
	1	98	68.1
	2	22	15.3
	3	12	8.3
	4	9	6.3
	5	3	2.1
	Total	144	100

Abbreviation: HRV, human rhinovirus.

### Intratype Genetic Diversity

**Figure 5.**
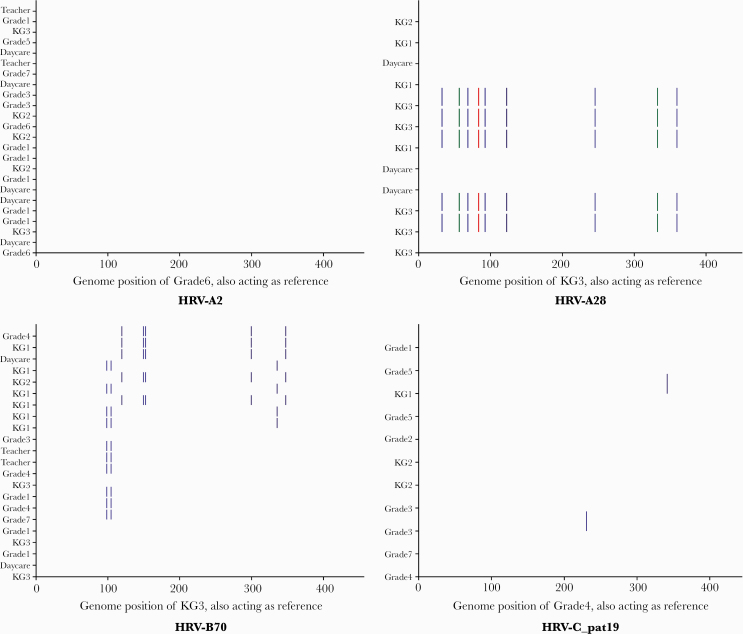

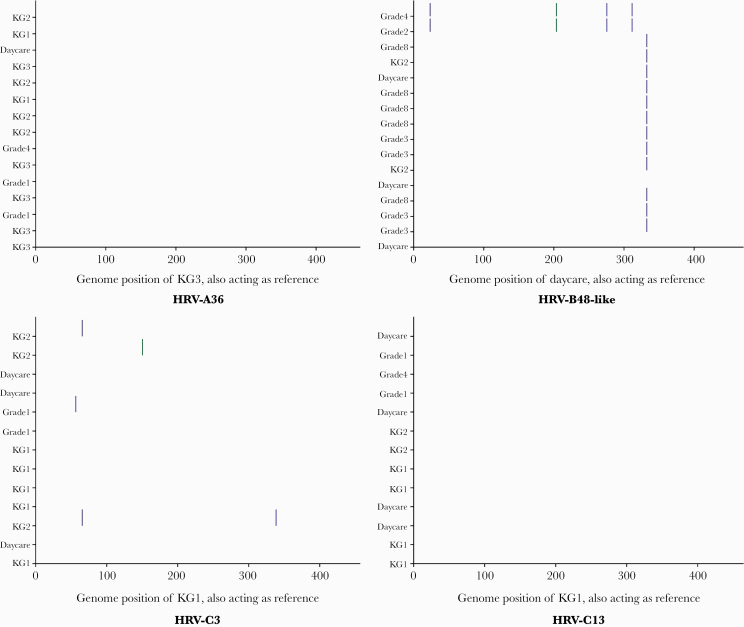
Genetic diversity of HRV genotypes with a frequency of >10. From the top left: A2, A28, A36, B48-like, B70, C_pat19, C3, and C13 (bottom right). Nucleotide substitutions are demonstrated by a colored bar. A substitution to an “A” is indicated by green, “T” by red, “G” by indigo, and “C” by blue bars. The sequences are labeled by grade of individual and ordered by date of sample collection with the genotype’s index sequence at the bottom (acting as a reference). Abbreviation: HRV, human rhinovirus.

Eight genotypes had ≥10 samples/sequences, and 5 of these occurred as a single variant throughout the study. The remaining 3, A28, B48, and B70, had >1 variant. A28 had 2 variants simultaneously observed. One variant was predominantly from KG1 (n = 4/5), while the second was heterogenous. For B48, the second variant was observed as a genotype recurrence in a subsequent school term. Genotype B70 had the highest diversity, with 3 variants observed within 1 school term. The first B70 variant (13 samples) occurred across lower and upper primary as well as teaching staff, the second (4 samples) was first observed 49 days after the genotype’s overall index sequence (all samples from KG class 1), and the third (5 samples) was first observed 69 days after the genotype index sequence, with 4/5 samples coming from the lower primary. No individual had >1 variant of the same genotype (Figure 5). Genotype-specific ML trees of these 3 genotypes showed variant-associated phylogenetic clustering, supported by bootstrap values >70%. Only 2 phylogenetic clusters were associated with grade: K1 (n = 4/5 samples from KG3) in A28 and K2 (n = 4 samples from KG1) in B70 (Supplementary Figure 1).

### HRV Among Teaching Staff

Thirty-two samples were collected from teachers, of which 5 (15.6%) were HRV positive from 4 individuals. Three HRV genotypes were identified: A2 (n = 2), B70 (n = 2), and B99 (n = 1). The A2 and B70 genotypes were detected in teachers several days after their initial detection in the student population. The B99 sample from a teacher was the only case of this genotype identified during the entire study period, suggesting that they acquired the infection from outside the school setting, and no onward transmission was observed.

## DISCUSSION

In this coastal Kenya school study, we found that HRV occurs year-round in line with studies in this location among symptomatic individuals within the KHDSS area (11%–23%) [31, 32, 38]. HRV was detected across all age groups, with the highest proportion in the <5-year-olds and the lowest proportion in older age groups (≥18 years), in agreement with previous findings [20, 39, 40]. A proportion of the HRV-positive children (14%) identified another household member as having ARI-like symptoms, suggesting transmission at the household level that might contribute to transmission at school (or vice versa).

All HRV species were found in circulation throughout the year. HRV-A circulated widely (53%), more than -B (18%) and -C (29%), in contrast to previous reports where HRV-A and HRV-C co-dominated [31, 32, 38]. However, a similar occurrence of HRV species was reported in the first 2 years of aboriginal and nonaboriginal Australian children [41]. There was considerable HRV diversity, with almost one-third of all known HRV genotypes detected. HRV infections were comprised of single genotype occurrences observed in a single sample/individual, as well as frequent genotypes affecting numerous pupils across several classes. This pattern has been observed elsewhere [42], and it is not clear whether this is due to varying serotype infectivity [43] or other epidemiological factors.

Numerous genotypes co-circulated in every school term, implying that no particular genotype predominates at any given time period. Previous studies have shown that contemporary HRV infections in a given population are characterized by numerous genotype-specific “mini-epidemics” [44] and that up to 30 genotypes circulate simultaneously in a given geographical area [45]. No frequent genotype was limited to 1 class, suggesting heterogeneous mixing and transmission within the school. However, 4 frequent genotypes and 1 variant of B70 were observed only in the lower primary, an indication of social clustering. Genotype recurrence in a subsequent school term was observed in 9 genotypes. Although it is not clear whether the study design missed samples between 2 genotype occurrences, the infrequency of genotype recurrence is possibly a reflection of herd immunity to specific types within the school/local community or a reflection of random introductions into the school/local community. Frequent genotypes in the school persisted for about a month on average. This is a shorter period than that observed across the KHDSS (a larger geographical scope) during an earlier outpatient surveillance study [38]. This is probably due to increased transmission (steered by high contact rates among school-going children), resulting in a shorter-duration epidemic.

The younger age groups exhibited high rhinovirus diversity, as they had more HRV re-infections. No individual was re-infected with the same genotype, further evidence of serotype-specific immunity to HRV lasting ≥1 year [46].

We demonstrate the occurrence of intragenotype variants and associated phylogenetic clusters, which were either separate rhinovirus introductions or diversification of a single variant after introduction, forming as a result different transmission clusters. This observation highlights the benefit of sequence data over serology to study viral transmission dynamics. The numerous HRV genotypes, sparse sampling of ARIs, and minimal resolution from partial short sequences obtained here did not allow for transmission inferences (due to insufficient within-type variation).

An outpatient health facility located at the same location as the school reported in this study was recruited into an ARI surveillance study from December 2015 to November 2016, 5 months before onset of the school surveillance [27]. This outpatient clinic is within 4 km from the school. A detailed analysis of molecular epidemiology of HRV for samples collected at this outpatient clinic has been reported elsewhere [38]. Although not a primary objective of this study, we compared the diversity of HRV infections between the 2 study periods. We observed 12 common genotypes in the 2 studies: A13, A20, A28, A31, A46, A54, A78, A101, B42, C6, C11, and C19. However, only 1 genotype was frequent in both periods: A101 (Supplementary Figure 2). Our comparison of HRV diversity between a school setting and clinical cases in a health facility within the same geographical location and 2 consecutive seasons showed only 1 frequent genotype present in both studies. This is an indication that HRV diversity within a community varies widely over time, as previously observed [42]. It is not definite what drives the exchange of common rhinovirus genotypes. The rapid turnover and coexistence of genotypes and variants might be determined by immunologically mediated selection processes or other nonselective epidemiological processes.

Our study had some limitations. First, the dichotomy in the number of samples collected weekly in lower vs upper primary posed a challenge when comparing the 2 groups. Second, weekly sampling of only symptomatic persons will likely have resulted in missed HRV infections, impairing the overview of HRV dynamics. In addition, the study failed to successfully amplify and sequence nearly 18% of HRV-positive samples. Failure was not correlated with viral load and may have been caused by variability in primer-annealing sites resulting in mismatches. This may have resulted in missed genotypes or subvariants.

This study provides improved knowledge of the diversity and temporal characteristics of HRV in a school setting, reinforcing the notion that schools are a focal point in understanding HRV transmission in the community. The effect of numerous individuals in close contact enabling HRV transmission is evident. In addition, we see that infections could be linked to transmission events occurring outside the school setting, that is, in the household setting. The contemporary inclusion of different population structures (eg, schools, households, health centers) in studying HRV dynamics will improve our understanding of HRV epidemiology in communities. Future studies should focus on whole-genome sequencing to fully elucidate transmission clusters.

## Supplementary Data

Supplementary materials are available at *Open Forum Infectious Diseases* online. Consisting of data provided by the authors to benefit the reader, the posted materials are not copyedited and are the sole responsibility of the authors, so questions or comments should be addressed to the corresponding author.

ofaa385_suppl_Supplementary_Figure_1A

ofaa385_suppl_Supplementary_Figure_1B

ofaa385_suppl_Supplementary_Figure_1C

ofaa385_suppl_Supplementary_Figure_2A

ofaa385_suppl_Supplementary_Figure_2B

ofaa385_suppl_Supplementary_Material
