# Supplementary figures and images for: Molecular Epidemiology of Human Rhinovirus From 1-Year Surveillance Within a School Setting in Rural Coastal Kenya
Source: Open Forum Infect Dis. 2020 Aug 27;7(10):ofaa385. doi: 10.1093/ofid/ofaa385 (PMC7568438; doi:10.1093/ofid/ofaa385)

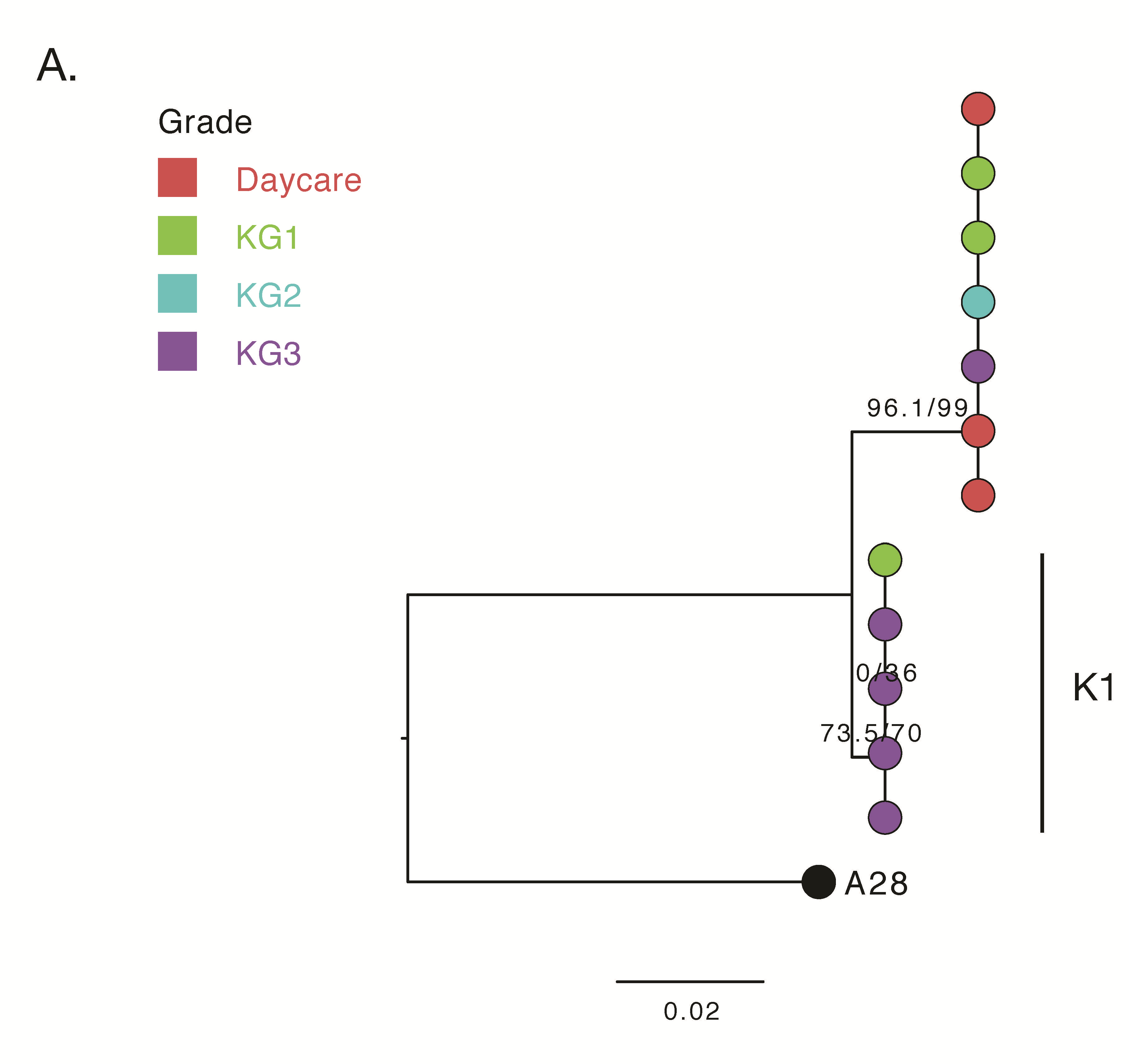

Supplement: ofaa385_suppl_Supplementary_Figure_1A [file ofaa385_suppl_supplementary_figure_1a.png]

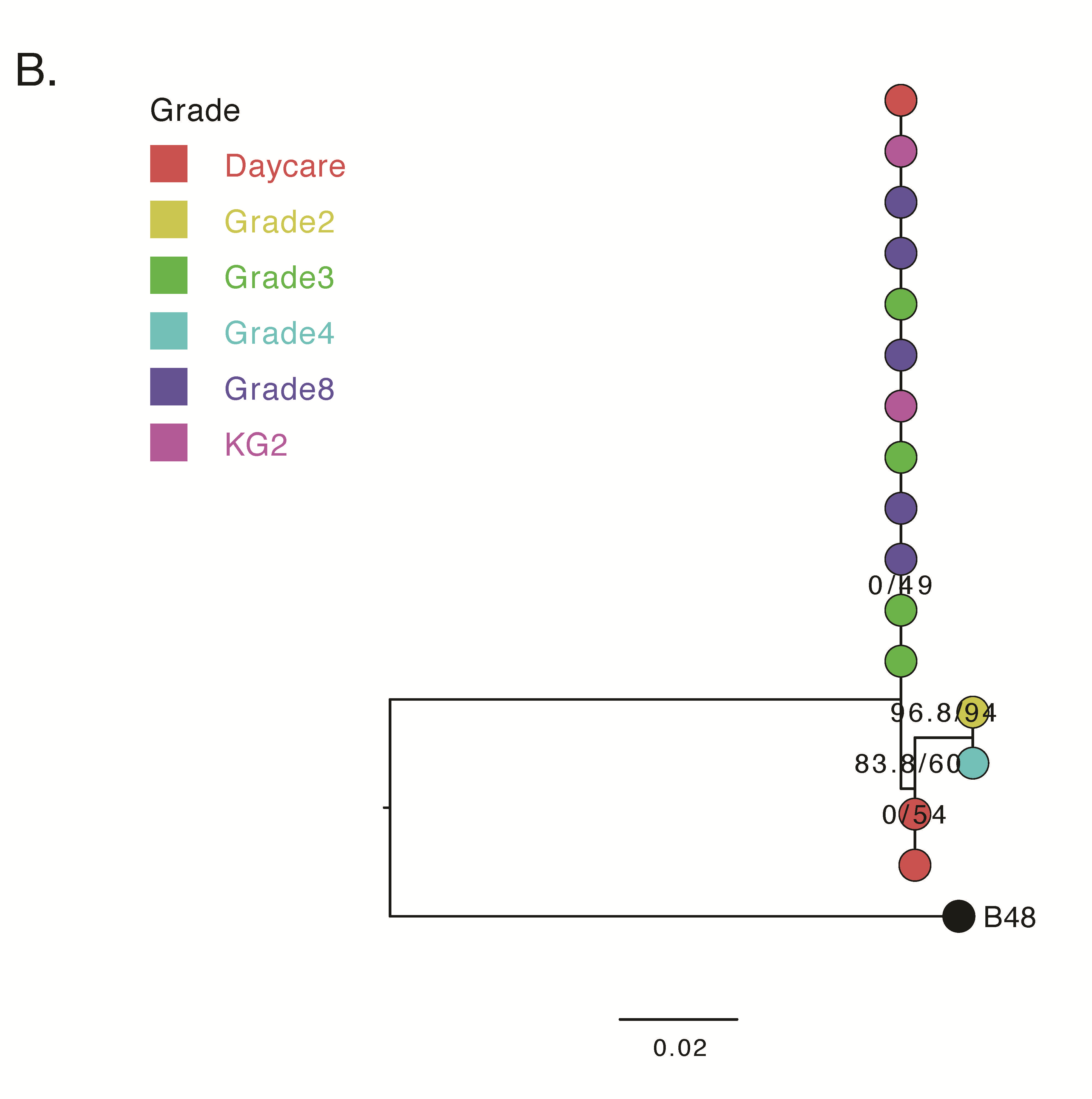

Supplement: ofaa385_suppl_Supplementary_Figure_1B [file ofaa385_suppl_supplementary_figure_1b.png]

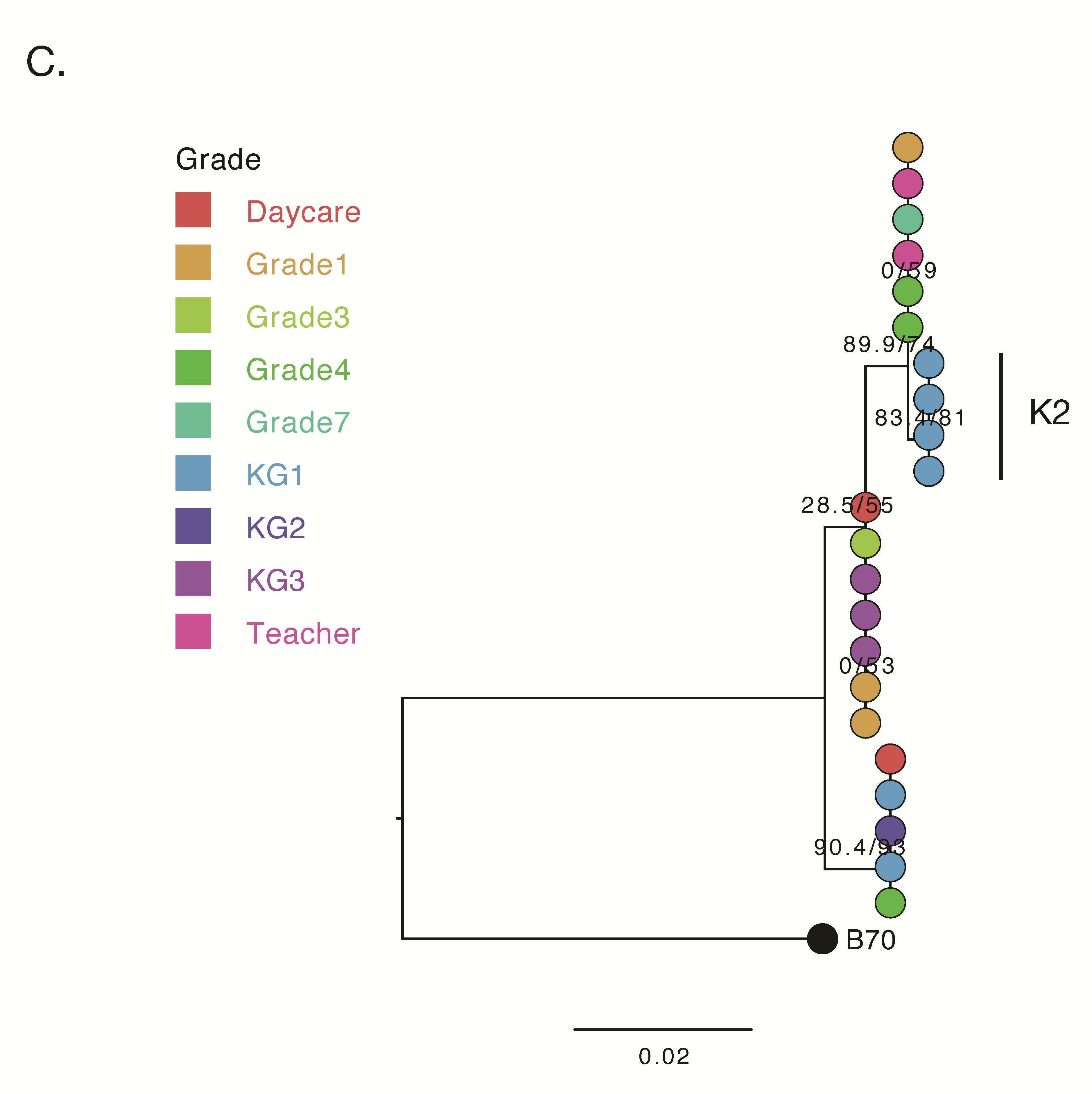

Supplement: ofaa385_suppl_Supplementary_Figure_1C [file ofaa385_suppl_supplementary_figure_1c.png]
