# Supplementary material for: Molecular Epidemiology of Human Rhinovirus From 1-Year Surveillance Within a School Setting in Rural Coastal Kenya
Source: Open Forum Infect Dis. 2020 Aug 27;7(10):ofaa385. doi: 10.1093/ofid/ofaa385 (PMC7568438; doi:10.1093/ofid/ofaa385)
Supplement: ofaa385_suppl_Supplementary_Material [file ofaa385_suppl_supplementary_material.docx]

**Supplementary Materials: Tables and Figure Legends**

**Supplementary Table 1: Detection of other virus targets among HRV positive and negative samples.**

|  | HRV positive samples n=307 | | HRV negative samples n=1552 | |
| --- | --- | --- | --- | --- |
| Target virus | **Frequency** | **Percentage** | **Frequency** | **Percentage** |
| Parainfluenza viruses (PIV) | **8** | **30.8** | **36** | **31.3** |
| PIV 1 | 0 | 0.0 | 0 | 0.0 |
| PIV 2 | 1 | 3.8 | 11 | 9.6 |
| PIV 3 | 6 | 23.1 | 15 | 13.0 |
| PIV 4 | 1 | 3.8 | 9 | 7.8 |
| PIV 2/ PIV 3 | 0 | 0.0 | 1 | 0.9 |
| Human coronaviruses (HCoV) | **4** | **15.4** | **28** | **24.3** |
| HCoV-OC43 | 3 | 11.5 | 8 | 7.0 |
| HCoV-NL63 | 1 | 3.8 | 16 | 13.9 |
| HCoV-E229 | 0 | 0.0 | 4 | 3.5 |
| Influenza viruses (Flu) | **3** | **11.5** | **12** | **10.4** |
| Flu-A | 0 | 0.0 | 3 | 2.6 |
| Flu-B | 3 | 11.5 | 7 | 6.1 |
| Flu-C | 0 | 0.0 | 0 | 0.0 |
| Flu-A/ Flu-B # | 0 | 0.0 | 1 | 0.9 |
| Flu-A/ Flu-B/ Flu-C # | 0 | 0.0 | 1 | 0.9 |
| Respiratory syncytial virus (A and B) | **1** | **3.8** | **8** | **7.0** |
| RSV-A | 1 | 3.8 | 7 | 6.1 |
| RSV-B | 0 | 0.0 | 1 | 0.9 |
| Human metapneumovirus (HMPV) | 6 | 23.1 | 12 | 10.4 |
| Adenovirus | 3 | 11.5 | 9 | 7.8 |
| HCoV/ PIV # | 0 | 0.0 | 3 | 2.6 |
| Adenovirus/ HMPV # | 0 | 0.0 | 2 | 1.7 |
| RSV / PIV # | 1 | 3.8 | 2 | 1.7 |
| Adenovirus/ PIV # | 0 | 0.0 | 1 | 0.9 |
| Adenovirus/ HCoV # | 0 | 0.0 | 1 | 0.9 |
| Flu / HMPV # | 0 | 0.0 | 1 | 0.9 |
| Total | **26** | **100** | **115** | **100** |
| # Co-detections | | | | |

**Supplementary Table 2: Limits of intra-type pairwise genetic distances of Kilifi school sequences to HRV prototype strains**

| Type | Accession number of prototype strain | Number of sequences | Upper limit p-distance |
| --- | --- | --- | --- |
| A1 | D002390.1 | 7 | 0.093 |
| A10 | DQ4734980.1 | 8 | 0.071 |
| A101 | GQ4150510.1 | 5 | 0.044 |
| A13 | FJ4451160.1 | 3 | 0.099 |
| A2 | X023160.1 | 24 | 0.102 |
| A20 * | FJ4451200.1 | 6 | 0.118 |
| A23 | DQ4734970.1 | 2 | 0.074 |
| A28 | DQ4735080.1 | 12 | 0.102 |
| A31 | FJ4451260.1 | 1 | 0.104 |
| A34 | DQ4735010.1 | 1 | 0.093 |
| A36 | DQ4735050.1 | 15 | 0.091 |
| A46 | DQ4735060.1 | 6 | 0.071 |
| A47 | FJ4451330.1 | 7 | 0.085 |
| A49 | DQ4734960.1 | 3 | 0.102 |
| A51 | FJ4451360.1 | 2 | 0.088 |
| A54-like * | FJ4451380.1 | 5 | 0.107 |
| A59 | DQ4735000.1 | 2 | 0.088 |
| A76 | DQ4735020.1 | 2 | 0.082 |
| A78 | EF1734180.1 | 3 | 0.102 |
| A8 | FJ4451130.1 | 6 | 0.085 |
| A80-like* | FJ4451560.1 | 1 | 0.137 |
| A82 * | DQ4735090.1 | 7 | 0.113 |
| A9 | FJ4451770.1 | 5 | 0.077 |
| A94 | EF1734190.1 | 1 | 0.102 |
| B42 | FJ4451300.1 | 1 | 0.073 |
| B48-like * | DQ4734880.1 | 16 | 0.154 |
| B70 | DQ4734890.1 | 22 | 0.09 |
| B79 | FJ4451550.1 | 2 | 0.051 |
| B86-like * | FJ4451640.1 | 2 | 0.121 |
| B97-like * | FJ4451720.1 | 2 | 0.118 |
| B99 | FJ4451740.1 | 1 | 0.076 |
| C_pat19 | FJ5980960.1 | 11 | 0.047 |
| C_pat21 | FJ6157370.1 | 1 | 0.061 |
| C10 | GQ3237740.1 | 6 | 0.05 |
| C11 | EU8409520.1 | 1 | 0.087 |
| C13 | EU0817950.1 | 13 | 0.041 |
| C16 | EU0818080.1 | 1 | 0.047 |
| C19 | EU6978500.1 | 2 | 0.055 |
| C21 | EU7523770.1 | 1 | 0.073 |
| C25 | EU7524270.1 | 3 | 0.105 |
| C27 | GQ2231220.1 | 1 | 0.026 |
| C3 | EF1860770.1 | 13 | 0.052 |
| C31 | GU2943800.1 | 7 | 0.079 |
| C36 | EF0772560.1 | 4 | 0.093 |
| C40 | EU0818020.1 | 1 | 0.064 |
| C53 | MF7753670.1 | 5 | 0.006 |
| C6 | EF5823870.1 | 3 | 0.061 |
| * Do not conform to proposed VP4/2 thresholds. This has been documented for genotypes A78 (intra-clade upper limit of 0.153), A82 (intra-clade upper limit of 0.135) and A20 (intra-clade upper limit of 0.119) ^22^. | | | |

**Supplementary Figure legend**

**Supplementary Figure 1:** Genotype-specific Maximum Likelihood trees of (A)HRV-A28, (B) HRV-B48 and (C) HRV-B70. Bra*n*ch labels indicate bootstrap values. The scale bar*s* *represent* nucleotide substitutions per site. The tip shapes are colored by the school class of the individual. The black tips represent the prototype sequence of respective strain. There are two statistically significant clusters by grade: K1 for HRV-A28 and K2 for HRV-B70.

**Supplementary Figure 2**: Genotype distribution between the school and the Junju outpatient clinic. (A). Phylogenetic analysis of sequences from the two studies. The tips are colored by study site. (B). A table with genotype frequencies from both studies. Only 12 genotypes were present in both studies.
